# Associations Between Knowledge of Health Conditions and Sugar-Sweetened Beverage Intake Among US Adults, 2021

**DOI:** 10.3390/nu16244317

**Published:** 2024-12-14

**Authors:** Jessica R. Hunter, Reena Oza-Frank, Sohyun Park, Ann Goding Sauer, Janelle P. Gunn

**Affiliations:** 1 Oak Ridge Institute for Science and Education (ORISE) Research Participation Program, 1299 Bethel Valley Rd, Oak Ridge, TN 37830, USA; ugt7@cdc.gov; 2 Centers for Disease Control and Prevention (CDC), 4770 Buford Highway, NE, Atlanta, GA 30341, USA; fqj7@cdc.gov (R.O.-F.); geo7@cdc.gov (S.P.); ftx0@cdc.gov (A.G.S.)

**Keywords:** added sugars, sugar-sweetened beverages, US adults, health conditions, knowledge, behavior, sociodemographic characteristics

## Abstract

Background: Frequent consumption of sugar-sweetened beverages (SSB) is associated with an increased risk of some health outcomes. Objective: We investigated the relationships between knowledge of health risks related to SSB and SSB intake among adults. Methods: This cross-sectional study utilized data from the 2021 SummerStyles survey. There were 4022 US adult participants (≥18 years). The outcome variable was SSB intake (none, >0 to <1, 1 to <2, or ≥2 times/day). The exposure variables were knowledge of the association between SSB and seven health conditions. Statistical analyses included seven multinomial regressions to estimate adjusted odds ratios (AOR) for the consumption of SSB according to knowledge of SSB-related health risks after controlling for sociodemographics. Results: Overall, about 30% of adults consumed SSB ≥ 2 times/day. While most adults identified SSB-related conditions such as weight gain (84.0%), diabetes (78.4%), and cavities (74.2%) as being related to drinking SSB, fewer adults recognized related conditions, such as some cancers (23.9%), high cholesterol (28.4%), heart disease (33.5%), and high blood pressure (37.8%). Knowledge of any of the health conditions was not significantly associated with consuming SSB ≥ 2 times/day compared to non-SSB consumers. Conclusions: Knowledge of SSB-related health conditions varied by sociodemographics but was not associated with high SSB intake. Future studies could explore other factors beyond knowledge that may influence adults’ high SSB intake.

## 1. Introduction

Excessive added sugars in the American diet are a significant public health concern [1,2]. Sugar-sweetened beverages (SSB) are the primary contributors of added sugars in the United States [1]. Frequent intake of SSB is linked to health consequences such as weight gain, cavities, type 2 diabetes (T2D), high cholesterol, high blood pressure, cardiovascular disease (CVD), and some cancers [3,4,5,6,7,8,9]. For example, a meta-analysis found that those who consumed 1–2 servings/day had a 26% greater risk of developing T2D than individuals with none or <1 serving/month [6]. In another study, SSB consumers with high intake were 2.17 times more likely to develop high blood pressure when compared to low SSB consumers [8]. SSB are defined as liquids that consist of added sugars such as sucrose, high-fructose corn syrups, or fruit juice concentrates [1]. SSB include, but are not limited to, fruit drinks, regular sodas, sports drinks, sweetened waters, and sweetened coffee and tea drinks [1]. According to the Dietary Guidelines for Americans (DGAs) 2020–2025, it is recommended that added sugars are limited to less than 10 percent of the caloric intake per day for persons 2 years and older [1]. For example, for an adult consuming a 2000-calorie diet, less than 200 calories should be from added sugars. However, according to 2015–2018 National Health and Nutrition Examination Survey (NHANES) data, the average percentage of calories from added sugars is 12.2% or an average of 242.3 calories from added sugars [10]. The consumption of SSB remains high among US adults. Based on the 2017–2018 NHANES, 77.9% of non-Hispanic Black adults, 77.8% of Hispanic adults, and 65.3% of non-Hispanic White adults consumed SSB on a given day [11]. Generally, health knowledge has been shown to positively influence behavior among adults [12,13]. SSB knowledge has been associated with behavior change or the adoption of health-promoting habits [14,15]. A previous study conducted in 2014 among US adults reported that a lack of knowledge of the SSB-related health condition of heart disease was significantly associated with high SSB consumption (≥2 times/day), while no association was found between SSB intake and knowledge of weight gain, diabetes, cavities, high cholesterol, and hypertension [16]. However, other studies have found no associations between knowledge and SSB intake [17,18]. It is unknown whether the SSB-related health knowledge has changed in recent years or has impacted SSB intake. Thus, this study’s objectives were to follow up on a previous study using newer data to further investigate the relationship between knowledge and SSB intake while controlling for sociodemographic factors among US adults [16].

## 2. Materials and Methods

This cross-sectional study was conducted using data from the SummerStyles survey, which was administered by Porter Novelli in the summer of 2021. SummerStyles is an online survey of a panel sample of US adults (≥18 years of age), created to evaluate a range of health-related attitudes, conditions, behaviors, and knowledge concerning public health issues. Survey participants were selected from Ipsos’ KnowledgePanel^®^, a large-scale online panel that uses an address-based sampling method [19]. When appropriate, households are provided with internet access and a computer. Because personal identifiers were not present in the data submitted to the Centers for Disease Control and Prevention (CDC), this analysis was exempt from review by the CDC institutional review board.

The SummerStyles survey was administered to a subset of individuals who also completed the Porter Novelli SpringStyles survey in March and April 2021. The SpringStyles survey was sent to a random sample of 10,919 panelists (≥18 years); 6455 completed the survey (59% response rate). The SummerStyles survey was distributed to a random sample of the 5741 adults who completed the SpringStyles survey in June and July 2021. Altogether, 4085 participants completed the SummerStyles survey (71% response rate). The data were weighted based upon age, race/ethnicity, sex, metro status, household income, household size, education level, census region, and the parental status of children between the ages of 12 and 17 years to match with the 2019 American Community Survey.

**Figure 1:**
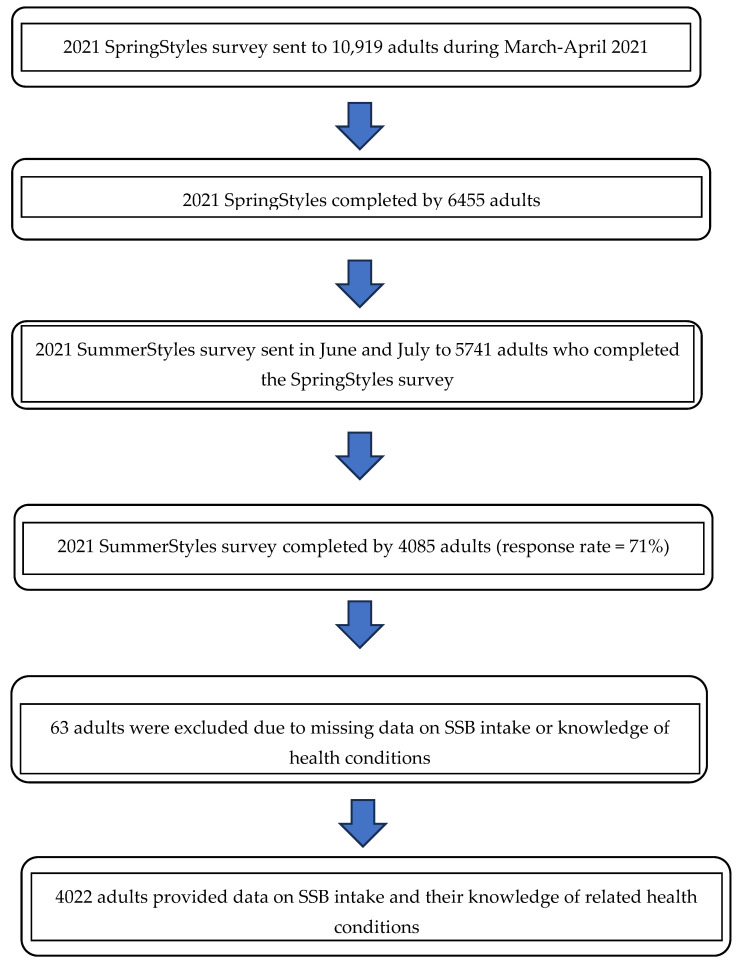
Analytic sample flow chart for SummerStyles survey among US adults, 2021. Outcome variable.

Sixty-three adults who completed the SummerStyles survey were excluded due to missing data on the outcome variable (i.e., SSBs: *n* = 45, 1.1%) or exposure variables (i.e., knowledge of the 7 SSB-related health conditions: *n* = 18, 0.4%), leaving an analytic sample of 4022 adults for the SSB and knowledge of health conditions objective (Figure 1). There were no significant differences observed in age, sex, race/ethnicity, weight status, marital status, annual household income, education level, and residential census area between the final analytic sample and the participants who were omitted.

The total SSB intake per day was the outcome of interest. The frequency of SSB intake was determined by the following 5 questions: (1) “During the past month, how often did you drink REGULAR SODA or pop that contains sugar? Do NOT include diet soda”; (2) “During the past month, how often did you drink COFFEE, including lattes, and TEA, including bottled tea, that was sweetened with sugar or honey? Do not include drinks with things like Stevia”; (3) “During the past month, how often did you drink SPORTS drinks such as Gatorade or Powerade? Do not include diet drinks”; (4) During the past month, how often did you drink ENERGY drinks like Red Bull, Monster, NOS, 5-Hour Energy, or Full Throttle? Do not include diet drinks”; and (5) “During the past month, how often did you drink sweetened fruit drinks, such as Kool-Aid, cranberry, and lemonade? Do not include 100% fruit juice”. For each question, the response options were none, less than once a week, 1–6 times/week, 1 time/day, 2 times/day, and 3 or more times/day. To calculate daily intake, less than once a week was first converted to 0.5 times/week and then converted to times per day (0.5 divided by 7 days); 1–6 times/week was converted to 3.5 times/week and then converted to times per day (3.5 divided by 7 days). To determine the frequency of total daily sugar-sweetened beverage consumption, we added the responses for the intake of regular soda, sweetened coffee/tea drinks, sports drinks, energy drinks, and fruit drinks. Four mutually exclusive categories were (0, >0 to <1, 1 to <2, or ≥2 times/day) established for total SSB intake based on the data distribution and a previous study [16].

### 2.1. Exposure Variables

Knowledge of 7 SSB-related health conditions constituted the main exposure variables. This was established by the following question: “Which of the following conditions do you think are related to drinking sugary drinks, such as regular sodas, fruit drinks (e.g., Kool-Aid, lemonade), sports or energy drinks (e.g., Gatorade, Red Bull), and sweetened teas?” Participants were then requested to select one or more health conditions: weight gain, diabetes, cavities, high cholesterol, heart disease, high blood pressure, and some cancers.

### 2.2. Covariates

For each covariate, mutually exclusive response categories were developed. Sociodemographic variables were age (18–24, 25–44, 45–64, and ≥65 years), sex, race/ethnicity (non-Hispanic White, non-Hispanic Black, Hispanic, or non-Hispanic other/multiracial), education level (≤high school, some college, and college graduate), and marital status (married/domestic partnership and not married). Not married included individuals who were widowed, divorced, separated, or never married. The annual household income was grouped into the following categories: <USD 35,000, USD 35,000–USD 74,999, USD 75,000–USD 99,999, or ≥USD 100,000. Based on the self-reported weight and height, the weight status was distributed among the following categories: underweight/healthy weight (BMI < 25 kg/m^2^), overweight (BMI 25–<30 kg/m^2^), or obesity (BMI ≥ 30 kg/m^2^) [20]. The census region of residence was categorized as Northeast, Midwest, South, and West [21].

### 2.3. Statistical Analysis

Chi-squared tests were used to analyze the bivariate relationships among SSB intake, knowledge of the 7 SSB-related health conditions, and sociodemographic characteristics, with a *p* value of <0.05 indicating statistical significance. Independent, multinomial logistic regression analyses were conducted to calculate adjusted odds ratios (ORs) and 95% confidence intervals (CIs) for the odds of consuming SSB ≥ 2 times/day among those who lacked knowledge of the SSB-related health condition versus those who had knowledge. While the outcome variable of SSB intake had 4 categories, adjusted ORs were presented for only the highest SSB intake group (≥2 times/day), compared with 0 times/day as the reference group. This approach was taken to compare the high- and no-SSB-intake groups. Separate logistic regression models were used for each health condition to avoid potential collinearity among the 7 health conditions and controlled for factors such as age, sex, race/ethnicity, education level, marital status, annual household income, weight status, and the census region of residence. Each statistical analysis was conducted using the Statistical Analysis Software (SAS; version 9.4; SAS Institute Inc., Cary, NC, USA), which incorporated procedures to account for the sample design by utilizing SURVEYFREQ and SURVEYLOGISTIC with WEIGHT statements.

## 3. Results

Of the 4022 participants within the analytic sample, 29.7% reported consuming sugar-sweetened beverages at least two times per day during the past 30 days (Table 1). SSB intake among the participants significantly varied by age, sex, race/ethnicity, education level, annual household income, and census region of residence (X^2^ tests, *p* < 0.05). In sociodemographic groups with overall significant differences, consuming SSB ≥ 2 times/day was most common among individuals aged 45–64 years, males, non-Hispanic White adults, those with ≤high school education, those with an annual household income of <USD 35,000, and those residing in the Northeast (Table 1).

Most adults were aware that weight gain (84.0%), diabetes (78.4%), and cavities (74.2%) are associated with consuming SSB. However, fewer adults identified that some cancers (23.9%), high cholesterol (28.4%), heart disease (33.5%), and high blood pressure (37.8%) are associated with consuming SSB. Furthermore, knowledge of the seven SSB-related health conditions significantly differed according to specific sociodemographic characteristics (X^2^ tests, *p* < 0.05). For instance, despite most adults identifying that weight gain is associated with SSB intake, their knowledge varied significantly by age, sex, race/ethnicity, education level, marital status, annual household income, and weight status. As another example, knowledge that high blood pressure is associated with SSB intake significantly differed by age and education level (Table 2).

Based on the bivariate analyses, SSB intake significantly differed according to the knowledge that weight gain and diabetes are related to SSB intake (X^2^ tests, *p* < 0.05, Table 3). However, the findings from the multinomial logistic regression analyses indicated that knowledge of any of the health conditions was not significantly associated with consuming SSB ≥ 2 times/day when compared to non-SSB consumers after controlling for covariates (Table 3).

## 4. Discussion

The present study, based on 2021 data, determined that while the majority of adults reported knowing that weight gain, diabetes, and cavities are associated with consuming SSB, a lower proportion of adults knew that high blood pressure, heart disease, high cholesterol, and some cancers are associated with consuming SSB. In comparison to a study conducted in 2014 among US adults, the SSB-related knowledge slightly increased from 2014 to 2021 among US adults [16]. In both this study and the one conducted in 2014, most adults knew that weight gain, diabetes, and cavities are associated with SSB intake (80.2%, 73.6%, 71.8% in 2014 and 84.0%, 78.4%, 74.2% in 2021), although the percentages were slightly higher for this study. Similarly, knowledge of health conditions such as high blood pressure, high cholesterol, and heart disease was less identified (33.0%, 24.1%, 31.5% in 2014 and 37.8%, 28.4%, 33.5% in 2021). According to the unadjusted analyses, knowledge that weight gain and diabetes are linked to drinking SSB was associated with SSB intake. After controlling for covariates, knowledge of any of the health conditions was not significantly associated with consuming SSB ≥ 2 times/day compared to non-SSB consumers in the present study. Findings from previous studies on the associations between knowledge of health outcomes related to SSB and SSB intake are mixed. A 2015 study determined, after controlling for covariates, that among 1000 Hispanic adults in the US, there was no significant association between SSB-related knowledge and SSB intake [22]. Contrary to our findings, another study found that knowledge of heart disease related to SSB consumption was significantly associated with lower SSB intake [16].

In the present study, approximately one in two US adults reported drinking SSB at least once a day and about one in three adults consumed them at least twice per day. In comparison to a previous study using 2014 SummerStyles data, the present study found that the prevalence of consuming SSB at least once a day (54.4%) was less than that found in 2014 (68.3) and the prevalence of high SSB intake (≥2 times/day) was lower in our study (29.7%) than in 2014 (37.8%) [16]. The decrease in high SSB intake could potentially be due to consumer awareness of added sugars. Of note, during this time period, the Food and Drug Administration made changes to the nutrition facts label to include added sugars in 2016 (effective in 2020), and there was implementation of public health education campaigns [23,24,25]. However, the high consumption of SSB is still a concern among US adults because of the excess calories that they add to the diet. While this study was not designed to assess causal reasons for high SSB consumption, the literature suggests that taste, increased availability, and mass media campaigns may influence the purchase and intake of SSB [26,27,28,29]. According to a 2021 study, during the COVID-19 pandemic, 20% of adults reported consuming more sweet foods and 10.6% of adults reported drinking more SSB [30]. As an example, drinking two 12 oz (355 mL) cans or two 16.9 oz (500 mL) bottles of regular soda per day could provide 276–420 kcal of excess calories or 78–116 g of added sugars each day [31].

Different types of knowledge interventions have been proposed, such as front-of-package labeling, warning labels, and an updated definition for “healthy” claims. Criteria for the proposed “healthy” claim include requirements that a food has to contain a specific amount of each food group (e.g., fruits, vegetables, grains) and cannot contain excessive saturated fat, sodium, or added sugars [32]. These public health strategies could be beneficial to consider in efforts to reduce added sugars. Front-of-package nutrition labeling policies have had an impact in reducing the sugar and calories in products through reformulation in several countries, including Chile, Ecuador, New Zealand, and the Netherlands [33]. In a 2016 study conducted among adults in Mississippi, it was determined that lower health literacy and nonuse of menu labeling was associated with higher SSB consumption [34]. Various studies have demonstrated the impact of nutrition education in improving knowledge and decreasing SSB intake among adults [35,36,37,38]. An experimental study conducted among adults in North Carolina found that exposure to SSB health warnings led to intent to lower SSB intake and evoked more attention, negative emotions, and thoughts about the consequences of SSB intake [14]. Another study conducted among 1413 US adults reported that warning labels that included health effects (obesity, diabetes, tooth decay) were considered as more effective than those without health effects, and warnings with nutrient disclosures (high in added sugars) resulted in higher perceived message effectiveness [39]. Additionally, there may be other interventions that could reduce the consumption of SSB, including educational campaigns that highlight alternative beverages and campaigns that facilitate improvements at the policy, environment, and community level. In Howard County, Maryland, there has been an effort to reduce SSB consumption through a multicomponent plan for district wellness policy implementation, collaboration with healthcare providers, and media campaigns. This resulted in a 29.7% decrease in ounces of regular soda sales, a 7.5% decrease in fruit drink sales, and a significant increase of 81.4 ounces in the sales of plain water per week [40].

In this study, there was a lack of association between knowledge of all SSB-related health conditions and high SSB intake among US adults. This could be because knowledge of SSB-related health conditions alone may be insufficient in eliciting behavioral changes related to SSB. As demonstrated in one study, factors related to behavior change include interest in consumption and perceived product healthfulness [36]. For example, the 2023 Food and Healthy Survey reported that one in three adults do not limit sugars because of taste preferences and belief that sugar intake is not a concern [41]. It is also possible that those with knowledge of the health risks of SSB do not change their SSB consumption because they may have misconceptions about their own risks for the development of chronic diseases [42]. Future studies could examine the barriers and facilitators of long-term behavioral changes and an individual’s understanding of long-term health consequences such as diabetes.

The current study has several limitations. First, the SummerStyles survey is a cross-sectional survey; therefore, causation cannot be determined. Second, due to the self-reported collection of the SummerStyles survey data, the responses may be subject to recall bias and social desirability response bias. Third, due to the limitations of the survey data, the depth or accuracy of health knowledge, cultural influences, taste preferences, accessibility and affordability of SSBs, and psychosocial factors were not available as variables to examine. Fourth, the research findings may not be generalizable to the entire US adult population, because the initial sample was selected from individuals willing to participate in the larger online-based knowledge panel. However, the data were weighted to be comparable to the US census. Finally, SSB intake was solely measured as a frequency; thus, the total volume of SSB consumed could not be computed.

## 5. Conclusions

To conclude, knowledge of SSB-related health conditions among US adults varied by health condition, ranging from 23.9% for some cancers to 78.4% for diabetes and 83.9% for weight gain. The majority of adults reported knowledge that SSB intake was associated with weight gain, diabetes, and cavities. Fewer adults identified high blood pressure, heart disease, high cholesterol, and some cancers as being related to consuming SSB. The absence of an association between knowledge of each SSB-related health condition and high SSB intake suggests that additional factors may influence consumption. Additional information on factors beyond knowledge and barriers to reducing SSB intake, such as marketing, alternative beverage availability, or habitual consumption, which may influence adults’ high SSB intake, could inform future public health strategies.

## Figures and Tables

**Table 1:** Characteristics of respondents and their associations with sugar-sweetened beverage (SSB) ^1^ intake among US adults participating in the SummerStyles survey, 2021 ^2^.

	All, Column % ± SE	By SSB Intake During the Past 30 Days, Row % ± SE				
Characteristic		0 Times/Day	>0 to <1 Time/Day	1 to <2 Times/Day	≥2 Times/Day	*p*-Value ^3^
Total (*n* = 4022)	100%	15.0 ± 0.7	30.5 ± 0.9	24.7 ± 0.8	29.7 ± 0.9	---
Age						
18–24 years	10.6 ± 0.8	12.6 ± 2.8	40.0 ± 4.2	25.2 ± 3.7	22.2 ± 3.6	<0.0001
25–44 years	34.9 ± 0.9	12.6 ± 1.1	32.4 ± 1.6	24.2 ± 1.4	30.8 ± 1.6	
45–64 years	33.4 ± 0.8	14.0 ± 1.0	29.3 ± 1.3	24.7 ± 1.2	32.0 ± 1.3	
≥65 years	21.1 ± 0.7	21.9 ± 1.4	24.8 ± 1.4	25.3 ± 1.4	28.0 ± 1.5	
Sex						
Male	48.5 ± 0.9	14.0 ± 0.9	29.5 ± 1.2	23.5 ± 1.1	33.0 ± 1.2	0.0032
Female	51.5 ± 0.9	15.9 ± 0.9	31.5 ± 1.3	25.9 ± 1.2	26.7 ± 1.2	
Race/ethnicity						
Black, non-Hispanic	11.6 ± 0.7	14.3 ± 2.5	35.3 ± 3.1	24.9 ± 2.7	25.5 ± 2.8	0.0110
Hispanic	16.2 ± 0.8	9.0 ± 1.4	34.7 ± 2.8	26.3 ± 2.5	30.0 ± 2.7	
Other, multiracial non-Hispanic	8.8 ± 0.6	14.4 ± 2.3	32.8 ± 3.1	25.0 ± 2.9	27.9 ± 3.0	
White, non-Hispanic	63.4 ± 1.0	16.8 ± 0.8	28.3 ± 1.0	24.2 ± 0.9	30.7 ± 1.0	
Education level						
High school or less ^4^	38.3 ± 1.0	13.1 ± 1.2	27.3 ± 1.5	22.7 ± 1.4	36.9 ± 1.7	<0.0001
Some college	30.0 ± 0.9	15.1 ± 1.1	30.9 ± 1.6	26.6 ± 1.5	27.4 ± 1.5	
College graduate	31.7 ± 0.8	17.2 ± 1.1	34.1 ± 1.4	25.4 ± 1.3	23.2 ± 1.2	
Marital status						
Married/domestic partnership	57.1 ± 1.0	15.1 ± 0.8	30.4 ± 1.0	24.9 ± 1.0	29.5 ± 1.0	0.9869
Not married ^5^	42.9 ± 1.0	14.9 ± 1.1	30.7 ± 1.5	24.4 ± 1.4	29.9 ± 1.5	
Annual household income						
<USD 35,000	19.9 ± 0.8	16.1 ± 1.8	24.9 ± 2.1	19.8 ± 1.9	39.2 ± 2.4	<0.0001
USD 35,000–USD 74,999	27.4 ± 0.9	11.7 ± 1.1	31.4 ± 1.8	27.7 ± 1.7	29.1 ± 1.6	
USD 75,000–USD 99,999	13.8 ± 0.6	13.2 ± 1.6	31.4 ± 2.3	24.4 ± 2.1	31.1 ± 2.3	
≥USD 100,000	38.9 ± 0.9	17.4 ± 1.0	32.5 ± 1.3	25.2 ± 1.2	24.8 ± 1.2	
Weight status ^6^ (*n* = 3946)						
Underweight/healthy weight	33.1 ± 0.9	16.5 ± 1.2	32.8 ± 1.7	24.2 ± 1.5	26.5 ± 1.5	0.1327
Overweight	32.3 ± 0.9	14.3 ± 1.1	29.0 ± 1.5	24.8 ±1.4	32.0 ± 1.5	
Obesity	34.5 ± 0.9	14.4 ± 1.1	29.4 ± 1.5	25.5 ± 1.4	30.6 ± 1.5	
Census region of residence						
Northeast	17.4 ± 0.7	19.1 ± 1.7	26.1 ± 1.9	21.8 ± 1.8	33.0 ± 2.0	<0.0001
Midwest	20.7 ± 0.7	13.7 ± 1.3	30.9 ± 1.8	28.3 ± 1.8	27.1 ± 1.8	
South	38.1 ± 0.9	15.0 ± 1.1	30.4 ± 1.5	22.3 ± 1.3	32.3 ± 1.5	
West	23.8 ± 0.8	13.2 ± 1.2	33.8 ± 1.9	27.5 ± 1.8	25.5 ± 1.7	

Abbreviations: SSB, sugar-sweetened beverage. ^1^ Frequency of SSB intake was calculated by adding 4 types of SSB (i.e., regular soda, fruit drink, sports/energy drink, and sweetened coffee/tea drink). ^2^ Weighted percentage may not add up to 100% due to rounding. ^3^ χ^2^ tests were used for each variable to examine differences across categories, and *p* value < 0.05 is considered statistically significant. ^4^ Includes General Educational Diploma (GED). ^5^ Widowed, divorced, separated, or never married. ^6^ Weight status was based on calculated body mass index (BMI; kg/m^2^): underweight/healthy weight, BMI < 25; overweight, BMI 25 to <30; obesity, BMI ≥ 30.

**Table 2:** Characteristics of respondents by knowledge of health conditions related to sugar-sweetened beverage (SSB) intake among US adults participating in the SummerStyles survey, 2021 ^1,2^.

	Knowledge of Health Conditions Related to SSB Intake (Answering Yes) ^3^, % ± SE						
Characteristic	Weight Gain	Diabetes	Cavities	High Cholesterol	Heart Disease	High Blood Pressure	Some Cancers
Total (*n* = 4022)	84.0 ± 0.8	78.4 ± 0.8	74.2 ± 0.8	28.4 ± 0.9	33.5 ± 0.9	37.8 ± 0.9	23.9 ± 0.8
Age							
18–24 years	78.7 ± 3.6 ^4^	77.0 ± 3.6	76.0 ± 3.6 ^4^	36.8 ± 4.1 ^4^	37.7 ± 4.1 ^4^	45.4 ± 4.2 ^4^	23.6 ± 3.6
25–44 years	80.8 ± 1.4	78.1 ± 1.5	76.5 ± 1.5	32.4 ± 1.6	37.0 ± 1.6	40.1 ± 1.6	26.2 ± 1.4
45–64 years	86.6 ± 1.0	79.8 ± 1.1	75.0 ± 1.3	23.5 ± 1.2	31.7 ± 1.3	37.2 ± 1.4	23.3 ± 1.2
≥65 years	87.5 ± 1.1	77.4 ± 1.4	68.1 ± 1.6	25.4 ± 1.4	28.5 ± 1.4	31.4 ± 1.5	21.1 ± 1.3
Sex							
Male	82.4 ± 1.1 ^4^	77.1 ± 1.1	71.7 ± 1.2 ^4^	28.3 ± 1.2	34.1 ± 1.2	38.7 ± 1.3	22.8 ± 1.1
Female	85.4 ± 1.1	79.7 ± 1.1	76.5 ± 1.2	28.5 ± 1.2	33.0 ± 1.3	37.0 ± 1.3	24.9 ± 1.2
Race/ethnicity							
Black, non-Hispanic	72.4 ± 3.1 ^4^	72.9 ± 2.9	66.8 ± 3.1 ^4^	26.6 ± 2.8	26.8 ± 2.8 ^4^	33.3 ± 3.0	19.3 ± 2.5
Hispanic	82.6 ± 2.2	78.5 ± 2.3	72.5 ± 2.5	29.9 ± 2.7	29.2 ± 2.5	37.6 ± 2.8	23.3 ± 2.4
Other, multiracial non-Hispanic	81.9 ± 2.7	81.7 ± 2.5	71.6 ± 3.0	32.9 ± 3.1	36.6 ± 3.2	43.5 ± 3.3	22.2 ± 2.7
White, non-Hispanic	86.7 ± 0.8	78.9 ± 0.9	76.3 ± 0.9	27.8 ± 1.0	35.4 ± 1.0	38.0 ± 1.0	25.2 ± 0.9
Education level							
High school or less ^5^	77.2 ± 1.6 ^4^	73.8 ± 1.5 ^4^	68.3 ± 1.6 ^4^	26.6 ± 1.5	25.9 ± 1.4 ^4^	34.1 ± 1.6 ^4^	18.2 ± 1.3 ^4^
Some college	86.8 ± 1.1	79.0 ± 1.4	74.8 ± 1.4	29.4 ± 1.6	34.7 ± 1.6	41.2 ± 1.7	25.6 ± 1.5
College graduate	89.5 ± 0.9	83.4 ± 1.1	80.7 ± 1.2	29.7 ± 1.3	41.6 ± 1.4	39.2 ± 1.4	29.3 ± 1.3
Marital status							
Married/domestic partnership	86.9 ± 0.8 ^4^	80.4 ± 0.9 ^4^	74.7 ± 1.0	26.6 ± 1.0 ^4^	33.2 ± 1.0	36.5 ± 1.1	23.7 ± 0.9
Not married ^6^	80.0 ± 1.4	75.7 ± 1.4	73.4 ± 1.5	30.9 ± 1.5	33.9 ± 1.5	39.7 ± 1.6	24.1 ± 1.4
Annual household income							
<USD 35,000	73.3 ± 2.3 ^4^	71.7 ± 2.2d	65.9 ± 2.3 ^4^	29.0 ± 2.3	26.5 ± 2.1 ^4^	35.5 ± 2.3	18.1 ± 1.8 ^4^
USD 35,000–USD 74,999	83.0 ± 1.5	75.2 ± 1.6	72.0 ± 1.7	27.9 ± 1.6	30.6 ± 1.7	38.2 ± 1.8	22.2 ± 1.5
USD 75,000–USD 99,999	84.1 ± 2.0	80.5 ± 2.0	74.5 ± 2.2	27.8 ± 2.2	33.7 ± 2.3	36.0 ± 2.4	23.5 ± 2.0
≥USD 100,000	90.0 ± 0.8	83.4 ± 1.0	79.8 ± 1.1	28.8 ± 1.3	39.1 ± 1.4	39.4 ± 1.4	28.2 ± 1.3
Weight status ^7^ (*n* = 3946)							
Underweight/healthy weight	80.5 ± 1.5 ^4^	77.3 ± 1.5	75.1 ± 1.5	28.8 ± 1.6	34.6 ± 1.6	35.8 ± 1.7	26.5 ± 1.5 ^4^
Overweight	86.2 ± 1.2	79.4 ± 1.3	72.1 ± 1.5	26.4 ± 1.4	32.6 ± 1.5	39.4 ± 1.6	24.0 ± 1.4
Obesity	85.4 ± 1.3	78.6 ± 1.4	75.1 ± 1.4	29.9 ± 1.5	33.2 ± 1.5	38.3 ± 1.6	21.8 ± 1.3
Census region of residence							
Northeast	83.1 ± 1.8	78.8 ± 1.9	73.2 ± 2.0	29.2 ± 2.0	35.3 ± 2.1 ^4^	38.2 ± 2.1	24.4 ± 1.8
Midwest	87.7 ± 1.4	81.1 ± 1.6	76.2 ± 1.8	28.5 ± 1.8	36.7 ± 1.9	40.0 ± 1.9	25.0 ± 1.7
South	82.1 ± 1.3	76.1 ± 1.4	72.1 ± 1.4	27.4 ± 1.4	29.8 ± 1.4	35.5 ± 1.5	22.3 ± 1.3
West	84.3 ± 1.5	79.5 ± 1.6	76.5 ± 1.7	29.4 ± 1.8	35.3 ± 1.9	39.6 ± 1.9	25.2 ± 1.7

^1^ Weighted percentage may not add up to 100% due to rounding. ^2^ χ^2^ tests were used for each variable to examine differences across categories. ^3^ Determined by the question, “Which of the following conditions do you think are related to drinking sugary drinks, such as regular sodas, fruit drinks (e.g., Kool-Aid, lemonade), sports or energy drinks (e.g., Gatorade, Red Bull), and sweetened teas?”. ^4^ *p* < 0.05 based on χ^2^ for test of overall difference in the category. ^5^ Includes General Educational Diploma (GED). ^6^ Widowed, divorced, separated, or never married. ^7^ Weight status was based on calculated body mass index (BMI; kg/m^2^): underweight/healthy weight, BMI < 25; overweight, BMI 25 to <30; obesity, BMI ≥ 30.

**Table 3:** Associations between sugar-sweetened beverage (SSB) ^1^ intake and knowledge of health conditions related to SSB intake among US adults participating in the SummerStyles survey, 2021.

Bivariate Analysis ^2^ (*n* = 4022)						Multinomial Logistic Regression Analysis ^3^ (*n* = 3946)
Knowledge of Health Conditions Related to SSB Intake ^4^	SSB Intake During the Past 30 Days, % ± SE					
	0 Times/Day	>0 to <1 Times/Day	1 to <2 Times/Day	≥2 Times/Day	*p*-Value ^5^	SSB Intake ≥2 Times/Day vs. 0 Times/Day, aOR (95% CI)
Weight gain						
Yes	14.4 ± 0.7	31.1 ± 0.9	25.7 ± 0.9	28.8 ± 0.9	0.0098	Reference
No	18.0 ± 2.2	27.7 ± 2.4	19.8 ± 2.0	34.4 ± 2.5		0.78 (0.55, 1.11)
Diabetes						
Yes	14.6 ± 0.7	31.0 ± 1.0	25.8 ± 0.9	28.5 ± 1.0	0.0132	Reference
No	16.4 ± 1.7	28.8 ± 2.0	20.7 ± 1.6	34.0 ± 2.0		0.97 (0.72, 1.32)
Cavities						
Yes	14.5 ± 0.7	31.1 ± 1.0	25.2 ± 0.9	29.2 ± 1.0	0.3163	Reference
No	16.6 ± 1.5	28.9 ± 1.8	23.4 ± 1.6	31.1 ± 1.8		0.83 (0.63, 1.10)
High cholesterol						
Yes	15.2 ± 1.2	30.9 ± 1.7	25.8 ± 1.6	28.1 ± 1.7	0.6813	Reference
No	14.9 ± 0.8	30.4 ± 1.0	24.3 ± 0.9	30.4 ± 1.0		1.08 (0.82, 1.41)
Heart disease						
Yes	15.8 ± 1.1	32.4 ± 1.5	24.1 ± 1.4	27.7 ± 1.4	0.2085	Reference
No	14.6 ± 0.8	29.6 ± 1.1	25.0 ± 1.0	30.7 ± 1.1		1.12 (0.88, 1.43)
High blood pressure						
Yes	15.2 ± 1.0	32.2 ± 1.5	23.2 ± 1.3	29.4 ± 1.4	0.3592	Reference
No	14.9 ± 0.8	29.6 ± 1.1	25.6 ± 1.0	29.9 ± 1.1		1.07 (0.83, 1.36)
Some cancers						
Yes	14.9 ± 1.2	32.8 ± 1.8	25.3± 1.7	27.0 ± 1.7	0.2649	Reference
No	15.1 ± 0.8	29.8 ± 1.0	24.5 ± 0.9	30.6 ± 1.0		1.03 (0.79, 1.33)

Abbreviations: CIs, confidence intervals; ORs, odds ratios; SSB, sugar-sweetened beverage. ^1^ SSB was calculated by adding 4 types of SSB (i.e., regular soda, fruit drink, sports/energy drink, and sweetened coffee/tea drink). ^2^ Weighted percentage may not add up to 100% due to rounding. ^3^ Values are adjusted ORs (95% CIs). The outcome variable was SSB intake, and the exposure variables were represented by knowledge of health conditions related to SSB intake. Because of potential collinearity issues among 7 exposure variables, 7 multinomial logistic regression models were fit to include each exposure variable separately and controlled for age, sex, race/ethnicity, education level, marital status, annual household income, weight status, and census region of residence. ^4^ Determined by the question, “Which of the following conditions do you think are related to drinking sugary drinks, such as regular sodas, fruit drinks (e.g., Kool-Aid, lemonade), sports or energy drinks (e.g., Gatorade, Red Bull), and sweetened teas?”. ^5^ χ^2^ tests were used for each variable to examine differences across categories.

## Data Availability

Data sharing is not applicable to this article.
